# Potential of Ferritin-Based Platforms for Tumor Immunotherapy

**DOI:** 10.3390/molecules27092716

**Published:** 2022-04-22

**Authors:** Xiaoling Xu, Kewei Tian, Xuefang Lou, Yongzhong Du

**Affiliations:** 1 Shulan International Medical College, Zhejiang Shuren University, Hangzhou 310015, China; ziyao1988@zju.edu.cn (X.X.); 11618079@zju.edu.cn (K.T.); 2 School of Medicine, Zhejiang University City College, Hangzhou 310015, China; 3 Institute of Pharmaceutics, College of Pharmaceutical Sciences, Zhejiang University, Hangzhou 310058, China

**Keywords:** ferritin, immunotherapy, dendritic cells, tumor cells, tumor-associated fibroblasts, M2 tumor-associated macrophages

## Abstract

Ferritin is an iron storage protein that plays a key role in iron homeostasis and cellular antioxidant activity. Ferritin has many advantages as a tumor immunotherapy platform, including a small particle size that allows for penetration into tumor-draining lymph nodes or tumor tissue, a unique structure consisting of 24 self-assembled subunits, cavities that can encapsulate drugs, natural targeting functions, and a modifiable outer surface. In this review, we summarize related research applying ferritin as a tumor immune vaccine or a nanocarrier for immunomodulator drugs based on different targeting mechanisms (including dendritic cells, tumor-associated macrophages, tumor-associated fibroblasts, and tumor cells). In addition, a ferritin-based tumor vaccine expected to protect against a wide range of coronaviruses by targeting multiple variants of SARS-CoV-2 has entered phase I clinical trials, and its efficacy is described in this review. Although ferritin is already on the road to transformation, there are still many difficulties to overcome. Therefore, three barriers (drug loading, modification sites, and animal models) are also discussed in this paper. Notwithstanding, the ferritin-based nanoplatform has great potential for tumor immunotherapy, with greater possibility of clinical transformation.

## 1. Introduction

Ferritin is a ubiquitous iron storage protein in living organisms and is considered to be a key protein [1,2], with dual roles in iron storage and antioxidation [3,4]. In 1937, Laufberger [5,6,7] isolated ferritin from horse spleen for the first time, and since then, it has also been found in humans, animals, plants, fungi, and bacteria. The amino acid sequences of ferritins isolated from different species are very different; mammalian ferritins are mainly composed of structurally similar heavy (H)-chain (21 kDa) and light (L)-chain (19 kDa) subunits [8,9,10]. The amino acid sequences of the H and L chains are 50% similar, but their functions are quite different [11]. The H-type subunit contains a ferrous oxidation center composed of seven conserved amino acids, H65, E27, E61, E107, Y34, E62, and Q141, and is mainly responsible for the rapid oxidation of ferrous ions. One ferrous oxidation center can simultaneously bind two ferrous ions. The L-type subunit lacks this ferrous oxidation center but contains a nucleation center, which is responsible for the slow oxidation of ferrous ions and subsequent iron nucleation and mineralization. The ratio of the H chain to the L chain is also different in different tissue types. For example, organs with high iron storage, such as the liver and spleen, have a large number of L-chain subunits, whereas organs with low iron storage, such as the heart and erythrocytes, have a large number of H-chain subunits, with a higher iron-binding efficiency [12,13]. The most commonly used ferritin is iron core-containing horse spleen ferritin of natural origin, which consists of 90% L-chain subunits and 10% H-chain subunits [14]. The second is a reconstituted human H-chain ferritin (hFTN), which has no iron core and is composed of 100% H-chain subunits.

Ferritin is an icosahedral-shaped protein cage composed of 24 subunits, with a relatively large molecular weight of approximately 450 kDa and an inner and outer diameter of 8 nm and 12 nm, respectively [15,16]. The inner core of ferritin is a cavity [17]; therefore, it can store 4500 Fe(III) atoms [18,19,20]. The main component of the iron core is a ferric oxyhydroxide–phosphate complex [20]. Ferritin nanocages possess a good self-assembly ability. Under extreme conditions, such as acidic pH, the quaternary structure of ferritin decomposes, and its subunits reassemble into cage-like structures once the pH returns to the physiological range [21]. Although the mechanism of its self-assembly is unknown, it has been shown that the protein shell can be opened by heating or adding a reducing agent; the complex of the inner core can be separated and then restored upon return to physiological conditions [15]. Iron-free ferritin nanocages with an 8-nm cavity, termed apoferritin, have been obtained [15]. The cavity can be used to encapsulate drug molecules or to biomineralize metal oxides. In 2005, Simek and Kilic [22] first reported the use of horse spleen ferritin nanocages to encapsulate the drug molecule doxorubicin. Subsequently, apoferritin-based drug delivery systems flourished and are widely used in the treatment of various diseases.

Ferritin has numerous advantages, which are described in Table 1, that place it ahead of other, more traditional materials in the clinical translation of nanodrug delivery systems for tumor therapy.

In recent years, ferritin has been an attractive protein nanoplatform for in vivo antigen delivery, antigen presentation, and immune stimulation. Ferritin nanoparticles (NPs) are self-assembled from 24 identical copies of ferritin subunits and have multiple regions that tolerate peptide insertions. Additionally, ferritin NPs have a well-defined spherical structure with an outer diameter of 12 nm, a size that is suitable for rapid penetration through tissue barriers and drainage to lymph nodes (LNs). Furthermore, ferritin NPs can be efficiently phagocytosed by dendritic cells (DCs) for antigen presentation in vitro [36]. Moreover, ferritin NPs exhibit remarkable thermal and chemical stability and are particularly amenable to recombination through a controlled disassembly/recombination process. This not only makes purification easier but also meets the stability requirements of an ideal vaccine.

Although ferritin-based drug delivery systems for tumor detection and therapy have been reviewed before, in this paper, we first summarized articles regarding ferritin-based nanoplatforms for tumor immunotherapy. We searched all for articles using different keywords in the database (Web of Science), including “nano” and “ferritin”, “liposome” and “ferritin”, “micelle” and “ferritin”, “nano” and “vaccine”, “liposome” and “vaccine”, and “micelle” and “vaccine”. Some articles suitable for this topic were supplemented during reading.

In this review, we introduce the design of ferritin-based nanoplatforms for tumor immunotherapy according to different targeting mechanisms (DCs, tumor cells, tumor-associated fibroblasts, and M2 tumor-associated macrophages (TAMs), among others). Finally, we provide a future outlook on ferritin-based vaccines for tumor immunotherapy.

## 2. Ferritin-Based Nanoplatforms for Cancer Immunotherapy

### 2.1. Targeting DCs

Cancer immunotherapy has emerged as an effective clinical modality for the treatment of malignant tumors that aims to enhance the patient’s antitumor immunity by activating tumor-specific CD8^+^ T cells and improving the suppressed tumor immune microenvironment [37]. However, there are three major problems. First, the ability of existing vaccines that carry tumor antigens to target APCs is weak [38]. A codelivery of immune adjuvants is often required to improve the antigen-presenting ability, but the use of adjuvants also introduces a series of toxic side effects, including fatal neurological syndrome [39], dialysis-related dementia, splenomegaly, and lymphoid follicle destruction and immunosuppression. Second, existing vaccines cannot alleviate immunosuppression in the tumor microenvironment. Consequently, some immune checkpoint inhibitors have been developed, but their overuse can activate the Fc in interleukin-17^+^CD4^+^ T helper 17 cells and cause systemic immune-related adverse events. Therefore, immune checkpoint inhibitor therapy is only suitable for patients with healthy immune function. Third, the production cost of monoclonal antibodies is high, resulting in an increased economic burden on patients.

**Figure 1:**
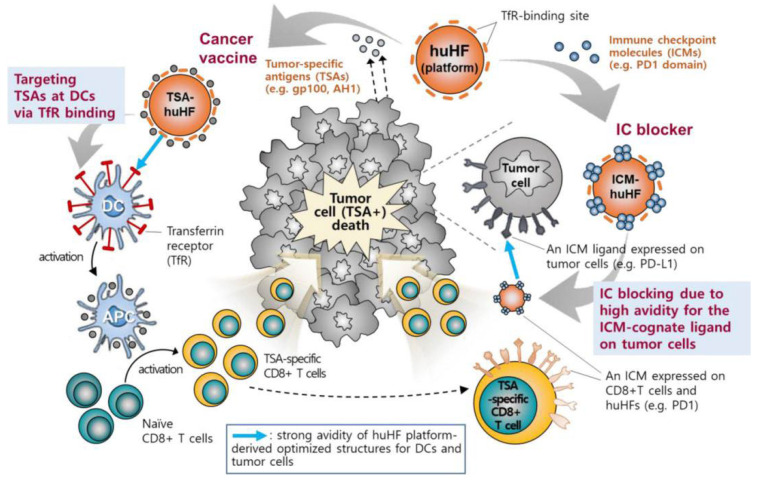
The mechanism of ferritin-based vaccines [41].

The application of hFTN-based vaccines provides a possibility to solve the above problems. This ferritin can not only be used to insert antigens through genetic engineering but can also promote antigen presentation and activate T-cell immunity by specifically binding to the overexpressed TfR on DCs. Lee et al. [40] evaluated the ability of four protein-based NPs with different origins, sizes, and shapes to target APCs in LNs. The particles included an *Escherichia coli* DNA-binding protein (Dps), the *Thermoplasma acidophilum* proteasome, the hepatitis B virus capsid, and hFTN. It was found that hFTN could be rapidly transported to and accumulated in large amounts in LNs. When the tumor antigen RFP was expressed on the surface of hFTN via genetic engineering, the ferritin induced RFP-specific immune responses in B16F10 melanoma and successfully inhibited tumor growth in tumor-bearing mice. In another study, Lee et al. [41] inserted the melanoma-specific antigen gp100 (KVPRNQDWL) into the N termini, C termini, and four interhelical loops (AB, BC, CD, and DE) of hFTN to construct six ferritin-based vaccines (Figure 1). Transmission electron microscopy (TEM) showed that the morphology of ferritin before and after the insertion remained basically unchanged, and the particle size was 12.5 to 14.7 nm. Among the six ferritins, CD-gp100-hFTN had the strongest affinity for TfR, whereas the affinity significantly decreased when gp100 was inserted into the BC loop. Both coincubation of DCs with Cy5.5-labeled ferritin and in vivo footpad injection showed that CD-gp100-hFTN effectively targeted LNs and presented the antigen on the DC surface. A lysosome-tracking experiment demonstrated that CD-gp100-hFTN could escape from endosomes to a cytosolic space and activate the NF-κB pathway in DCs, which resulted in augmented MHC-I-associated cross-presentation of the tumor-specific antigen. In vivo antitumor experiments showed that mice immunized with CD-gp100-hFTN exhibited the lowest tumor volume on day 21, with the maximum population of gp100-specific IFNγ^+^CD8^+^ T cells in splenocytes. Furthermore, the active region (G22 to V170) of PD-1 was inserted into the C terminus of the H-chain subunit of hFTN to construct a ferritin that could inhibit tumor immune checkpoints. After recombinant expression of the protein, the particle size was 22.02 ± 1.93 nm. Compared with that of anti-PD-L1 monoclonal immunoglobulin G, the dissociation constant increased from 255.10 ± 42.37 to 327.59 ± 89.45 nM, leading to enhanced tumor accumulation of the PD-1-presenting hFTN and efficient PD-L1 blockade. In vivo, PD-1-hFTN showed strong antitumor effects and reduced immune-related adverse events in CT26 and B16F10 tumor-bearing mice. Surprisingly, a combination therapy with PD-1-hFTN and CD-TSA-hFTN induced long-term antitumor immunity, preventing tumor recurrence and metastasis. In addition, Han et al. [42] inserted ovalbumin-derived OT-1 (SIINFEKL) and OT-2 (ISQAVHAAHAEINEAGR) antigen peptides into position 146 and the C terminus of ferritin, respectively, to construct antigen-delivery nanoplatforms OT-FPCN-L and OT-FPCN-C. In vitro, low concentrations of OT-FPCN-C and OT-FPCN-L showed no significant difference in their effects on CD8^+^ or CD4^+^ T-cell proliferation. However, in vivo, the ability of OT-FPCN-L to promote the proliferation of CD4^+^ T cells was weaker. The epitope loop structure carried by OT-FPCN-L was located in the middle of the protein sequence, and the ability of the inserted antigenic peptide to promote the maturation of DCs was weaker than that of the antigenic peptide inserted into the C terminus. Therefore, the site of insertion of an antigenic peptide or protein in ferritin should be carefully considered for in vivo applications.

There have been many studies regarding the display of exogenous peptides or proteins on the surface or in the cavity of ferritin using chemical modification or physical encapsulation as a vaccine platform, and good curative effects have been achieved. However, tumors are often affected by various factors that lead to mutations and the formation of new antigens. For neoantigens, the protein fusion step needs to be repeated, which is time-consuming and cannot meet the needs of clinically personalized antigen presentation. To establish a more flexible and efficient ferritin-based vaccine platform, Wang et al. [43] introduced the SpyTag/SpyCatcher (SC) combination technology. The SpyTag/SC system was developed based on the cleavage of the CnaB2 protein from *Streptococcus pyogenes*. CnaB2 is a domain isolated from the *S. pyogenes* fibronectin FbaB, which can be split into two parts, namely SpyTag (13 amino acid residues) and SC (116 amino acid residues). Aspartic acid in the SpyTag and lysine in the SC can spontaneously react to form covalent bonds under varying temperature (4–37 °C), pH (5–8) and buffer conditions. The study found that SpyTag and SC did not contain cysteine residues. Therefore, irrespective of whether SpyTag and SC are fused at the N terminus, C terminus, or in the middle of the protein, they can specifically bind and spontaneously form a heterozygous peptide bond. Therefore, the SpyTag/SC system can combine protein assembly and a genetically encoded chemical reaction and has important application potential as a monovalent/multivalent tumor vaccine platform. Wang et al. [39] first fused an improved version of the SC (amino acids 24–47 deleted from the N terminus) to the N terminus of ferritin to prepare SC–ferritin NPs. The fusion process did not affect the self-assembly ability of native ferritin, and SC–ferritin still had a smooth spherical cage structure, uniform size distribution (average particle size of 20.2 nm), and good stability (zeta potential of −24.5 mV). Subsequently, the purified SpyTag-E7 was mixed with SC–ferritin at different ratios. Because the SC and SpyTag mediate fast and efficient covalent binding, SpyTag-E7 and SC–ferritin quickly formed a ferritin displaying the E7 antigen. TEM showed a change from a smooth, spherical surface to a surface with prominent peaks, but the ferritin structure still maintained good dispersion and stability. Subcutaneous immunization of C57BL/6 mice revealed that ferritin could be rapidly and efficiently excreted into LNs and captured in vivo by DCs, especially by the CD8α^+^ population, to elicit a cytotoxic T-lymphocyte (CTL) response. Compared with soluble peptide antigens, ferritin NPs carrying the HPV16 oncogene E7 peptide antigen or an MC38 tumor-derived mutant neoantigen enhanced the antigen-specific CTL responses by 2–3-fold and significantly inhibited the growth of E7-related or MC38 tumors. Interestingly, a ferritin + E7 mixture induced little E7-specific CTL response, suggesting that the covalent binding of E7 to the ferritin surface, rather than simple physical mixing, is critical for vaccine efficacy. In addition, when the ferritin E7 (43–62)-based vaccine was used in combination with anti-PD-1, the antitumor effect was further enhanced, with 20% of the tested mice surviving for at least 80 days. This novel ferritin-based vaccine platform combines a single or multiple tumor-specific antigens, providing a new strategy for tumor vaccine construction.

Although highly effective vaccines for preventing pathogenic human papillomavirus (HPV) infection have been introduced for cervical cancer prevention, broadly protective and cost-effective approaches are still needed. The HPV minor capsid protein L2 is considered a promising replacement for the major capsid protein L1 because L2 is able to induce responses against a wider range of HPV types. However, a major limitation of L2 as a source of cross-neutralizing epitopes is that it is less immunogenic than L1 when assembled into VLPs. Meanwhile, existing HPV vaccines still require cryogenic storage and cannot benefit sick populations in underdeveloped countries. Therefore, Yang et al. [44] proposed the capsid protein L2 on the surface of ferritin, which can withstand high temperatures. The results showed that decoration with L2 did not interfere with the self-assembly of ferritin into an octahedral structure composed of 24 protomers. In guinea pigs and mice, ferritin NPs with the L2 antigen induced broad neutralizing antibody responses against 14 oncogenic and two non-oncogenic HPV types that persisted for at least 1 year. Taken together, the results of this study suggest that ferritin NPs hold promise as a powerful platform for HPV vaccines.

### 2.2. Targeting Tumor Cells

Innate immune cells (macrophages, DCs, and others), which play a major role in the host’s initial defense against pathogens, mediate the activation of the adaptive immune system through phagocytosis and antigen-presentation processes [45]. However, tumor cells specifically upregulate CD47 receptors, releasing a “don’t eat me” signal to avoid phagocytosis by innate immune cells. Studies have shown that blocking the CD47/SIRPα signaling pathway between tumor cells and phagocytes can increase the phagocytosis of tumor cells by innate immune cells, thereby enhancing the antitumor immune response [46]. Based on this, Lee et al. [47] engineered hFTN by decorating its surface with an SIRPα variant for binding to the CD47 receptor on the tumor cell. The recombinant ferritin (FH-SIRPα) could self-assemble into cage-like structures with an outer diameter of 16.6 ± 2.3 nm. Analysis of the binding kinetics of this ferritin to CD47 revealed an approximately 17–110-fold increase in the binding affinity of FH-SIRPα compared with that of the monomeric form of SIRPα. FH-SIRPα, tumor cells, and bone-marrow-derived macrophages/DCs were co-incubated to investigate phagocytosis. It was found that the FH-SIRPα treatment significantly enhanced the phagocytosis of tumor cells by innate immune cells compared with that in the untreated group. In vivo, FH-SIRPα treatment significantly enhanced effector T-cell immune responses in tumor-draining LNs, whereas no significant changes were observed in T-cell immune responses in the spleen. Cho et al. [48] compared the tumor immunotherapy effects of SIRPα-rich exosomes and ferritin and demonstrated that intratumoral injection of SIRPα-rich exosomes more effectively inhibited tumor growth, indicating that the blocking ability of the exosome-mediated CD47/SIRPα pathway was significantly stronger than that of ferritin at the same dose. The possible reason is that the density of SIRPα on the surface of exosomes was high, allowing aggregation into active clusters after binding to CD47 overexpressed on the surface of tumor cells and thereby promoting signal transduction.

**Figure 2:**
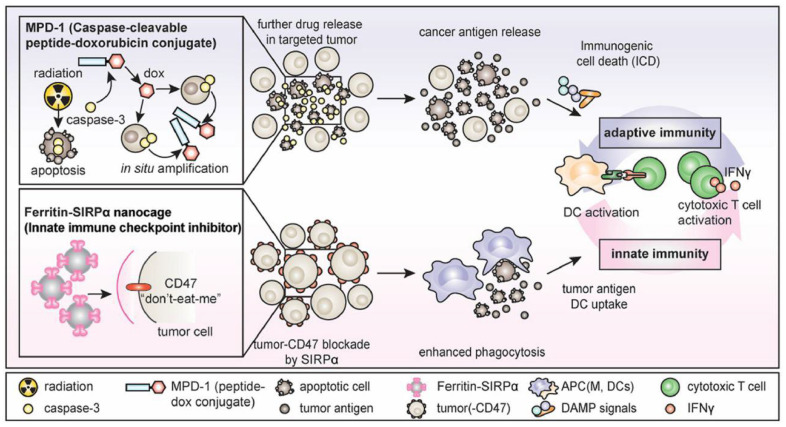
The combination of ferritin-SIRPα nanocages and caspase-cleavable peptide-doxorubicin conjugate [49].

SIRPα–ferritin nanocages have been confirmed as an antitumor agent in vitro and in vivo, but their therapeutic effect is still limited, which may be related to the single action pathway [25]. To further increase the exposure of antigens in the tumor immune microenvironment and enhance tumor-specific immunity, Lee et al. [49] combined SIRPα–ferritin with chemotherapy (Figure 2). The proposed strategy aimed to induce tumor immunogenic death using a doxorubicin prodrug to enhance the signal of phagocytosis. Subsequently, SIRPα–ferritin antagonized against CD47 for further enhanced tumor-specific immunity, achieving synergistic antitumor effects. In this study, an albumin-binding peptide–caspase-cleavable linker–doxorubicin conjugate was first synthesized, in which the albumin-binding peptide mediated the extension of the drug half-life to 106 h. Subsequently, the doxorubicin prodrug was cleaved by caspase-3 to release the antitumor drug doxorubicin, which induces tumor immunogenic death, significantly increases the levels of antigens and damage-related pattern molecules, and promotes lymphocyte infiltration into the tumor. At the same time, SIRPα-expressing ferritin induced massive phagocytosis of tumor cells by macrophages by blocking the SIRPα/CD47 pathway. Ultimately, the combination of SIRPα–ferritin and the doxorubicin prodrug eradicated colorectal cancer and elicited tumor-specific memory.

Similar to natural SIRPα, a PD-L1-binding peptide (CLQKTPKQC, with an affinity of 370 nM), which was developed by phage display technology, is expected to be used as a therapeutic alternative to antibodies, as it is an inexpensive PD-L1-targeting immune checkpoint molecule. Jeon et al. [50] used genetic engineering to construct ferritin nanocages with 24 PD-L1-binding peptides displayed on the outer surface, whereas the antitumor drug doxorubicin was loaded to the ferritin cavity for synergy between chemotherapy and immunotherapy against tumors. First, three ferritin constructs were designed, in which the PD-L1-binding peptide was linked to the N terminus (PpNF), C terminus (PpCF), and the loop between the fourth and fifth helices of short ferritin heavy chain (PpLF), respectively. TEM showed that all three constructs had complete cage structures, and dynamic light scattering (DLS) showed that the nanocage sizes of PpNF, PpLF, and PpCF were 24.0 ± 3.6 nm, 13.3 ± 2.6 nm, and 39.3 ± 9.3 nm, respectively. In vitro binding experiments showed that PpNF and PpCF could strongly bind to MDA-MB231 cells, and the binding was further enhanced if cells were pretreated with IFNγ, a potent inducer of PD-L1; however, PpCF showed a weaker binding than PpNF. By contrast, PpLF did not bind to MDA-MB231 cells, even with IFNγ treatment. An assessment of the binding kinetics showed that PpNF had a dissociation constant (*K_D_*) of 38 nM, whereas PpCF at concentrations up to 200 nM did not bind to PD-L1. In vivo distribution experiments showed that PpNF accumulated at tumor sites in larger amounts over a period of 24 h than unmodified ferritin without the PD-L1-binding peptide. Unlike PpCF, PpNF activated T-cell immune responses and significantly inhibited tumor growth. After combination with doxorubicin, the ferritin nanodelivery system PpNF(Dox) showed an even greater inhibitory effect on tumor growth. Meanwhile, after PpNF(Dox) treatment, mice lost little body weight, but there were no obvious side effects on hematological parameters, such as blood cell count or liver and kidney function.

### 2.3. Targeting Tumor-Associated Fibroblasts

T cell-mediated tumor immunotherapy is mainly aimed at increasing the number or activity of T cells and promoting the infiltration of existing T cells into tumor tissue. Existing anti-PD-1/anti-PD-L1 treatments are mainly aimed at increasing the number or activity of T cells without promoting T-cell infiltration. T-cell infiltration is still limited by the dense extracellular matrix (ECM) in tumor tissue. Studies have reported that the tumor has a dense outer shell, which is mainly composed of hyaluronic acid and collagen. Collagen forms a tight fibrotic network, and hyaluronic acid helps retain water, thereby increasing the interstitial fluid pressure in tumors and further impairing T-cell infiltration. The formation of a dense ECM is closely related to the secretion of various factors (collagens I, III, IV, and V, as well as VEGFA, EGF, FGF, HGF, and CXCL12) by tumor-associated fibroblasts in tumor tissue. Based on this, Zhen et al. [51] used hFTN as a nanocarrier loaded with the photosensitizer ZnF16Pc. To further construct nanocages, a tumor-associated fibroblast activation protein-specific binding fragment was covalently linked to the surface using bis(sulfosuccinimidyl) suberate as a crosslinker. The nanocages did not directly kill tumor cells but promoted T-cell infiltration by destroying the dense ECM in tumor tissue and inhibiting the secretion of CXCL12, thereby inhibiting tumor growth. Because only localized light irradiation was used, the nanocages caused little damage to healthy tissue and had good biosafety.

**Figure 3:**
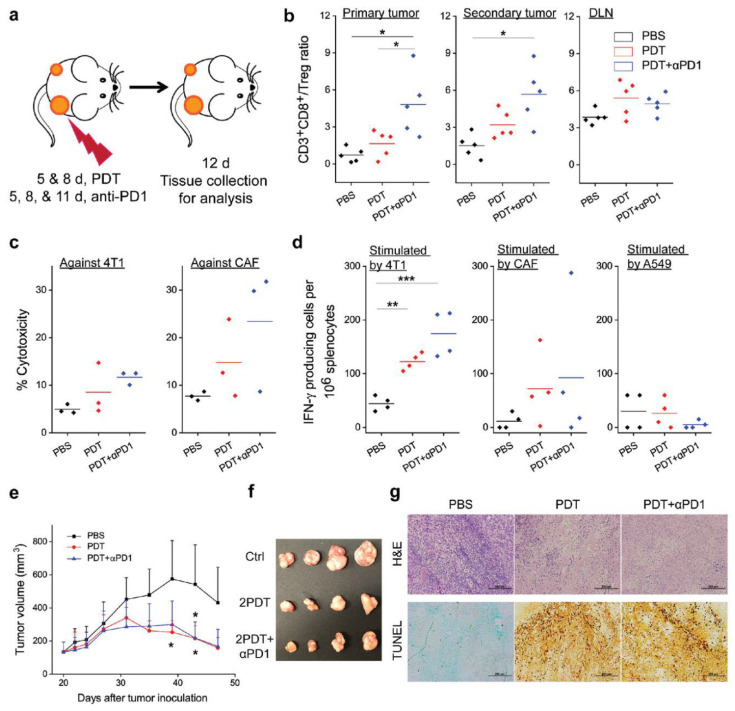
αFAP-Z@FRT induces anticancer and anti-CAF immunity [52]. (**a**) Schematic showing the treatment plan. (**b**) The ratios of CD3+CD8+ to Treg (CD3+CD4+FOXP3+) in primary tumor, secondary tumor, and tumor-draining lymph nodes (DLNs) were analyzed by flow cytometry. (**c**) Cell-specific cytotoxicity was assessed by propidium iodide (PI) staining followed by flow cytometry analysis. (**d**) IFN-γ-producing cells were quantified by enzyme-linked immune-absorbent spot analysis. (**e**) Growth curves of A549 tumors after adoptive cell transfer. (**f**) Photograph of A549 tumors taken on day 47 post A549 inoculation. (**g**) H&E and TUNEL staining, performed with A549 tumors taken on day 47. * *p* < 0.05; ** *p* < 0.01; *** *p* < 0.001.

On this basis, Zhou et al. [52] further investigated whether αFAP-Z@FRT could induce antigen-specific immunity and sustained tumor inhibition (Figure 3). The antitumor effect was investigated by eliminating CD4^+^ or CD8^+^ T cells in mice, and it was found that CD8^+^ cell depletion significantly accelerated tumor growth, which indicated that αFAP-Z@FRT elicited tumor-specific T-cell immune responses. In a bilateral 4T1 tumor model, αFAP-Z@FRT treatment not only effectively inhibited primary tumor growth on the treated side on day 23 but also reduced the contralateral, untreated tumor volume by approximately ninefold. Further combination of immune checkpoint inhibitors, such as an anti-PD-1 antibody (αPD-1) with αFAP-Z@FRT, significantly inhibited tumor growth and a rechallenge with viable 4T1 cells (25% of the animals completely rejected secondary tumors). ELISA analysis indicated that the average number of 4T1-specific effector T cells (per million splenocytes) was 38.0 in the PBS control group and increased to 108.5 in the αFAP-Z@FRT group and to 185.0 in the αFAP-Z@FRT and αPD-1 combination group. Considering CAF-specific effector T cells, there were 67.5 and 76.5 in the PDT and combination group, respectively, compared with 9.0 in the PBS control group. These results suggested that αFAP-Z@FRT elicited anti-CAF immunity while stimulating an immune response against cancer cells. Adoptive cell transfer showed that T cells from 4T1 tumor-bearing mice that treated with αFAP-Z@FRT retarded the growth of A549 tumors established in nude mice. Given that CAFs are present in almost all solid tumors and are genetically more stable than cancer cells, anti-CAF PDT may serve as a unique approach to antitumor vaccination.

### 2.4. Targeting M2 TAMs

TAMs are the main immune cells in the tumor microenvironment and play an important role in tumor growth and metastasis [53,54]. Tumor resident TAMs are usually of the M2 type. Therefore, repolarization of TAMs to the proinflammatory M1 type is a promising cancer treatment strategy. Toll-like receptor agonists, such as CpG oligodeoxynucleotides (CpGODNs), can induce tumor macrophage polarization. However, free CpGODNs cannot penetrate the cell membrane and are easily cleared by nucleases; therefore, in vivo application of this modality is still limited. Shan et al. [55] combined an M2 macrophage-targeting peptide with the N terminus of hFTN by genetic engineering, and a cationic peptide (CP) was fused to the C terminus of ferritin to construct specific ferritin nanocages (M2pep-rHF-CpG). The CP molecules displayed on the surface of ferritin significantly facilitated the encapsulation of CpGODNs in nanocages through electrostatic interactions and protected CpGODNs from nuclease degradation. The results of cellular uptake showed that M2pep-rHF-CpG selectively penetrated through the cell membrane of macrophages, and its uptake by B16F10 tumor cells was weak. The induction of a proinflammatory phenotype showed that the treatment of mouse RAW 264.7 cells with M2pep-rHF-CpG NPs increased the relative expression of M1 macrophage markers. The expression of M1 markers was positively correlated with the concentration of M2pep-rHF-CpG NPs. Enhanced fluorescence was observed in tumors in the M2pep-rHF-CpG-Cy7 group at 6, 12, and 24 h after intravenous administration, indicating an excellent tumor accumulation ability of the prepared nanocages. In vivo antitumor experiments showed that M2pep-rHF-CpG-Cy7 significantly inhibited the tumor growth, which may have been due to a significant increase in the M1/M2 ratio in the tumors, from 0.265 in the PBS-treated group to 0.541 in the NP-treated group. Thus, M2pep-rHF-CpG NPs can reprogram the tumor microenvironment, repolarize M2-type TAMs to M1-type TAMS, and inhibit tumor growth, which represents a novel approach for ferritin-based tumor immunotherapy.

### 2.5. Other Applications

The initiation, activation, and reactivation of T cells play an important role in tumor immunity, and this process is closely related to the PD-1/PD-L1 pathway in the tumor microenvironment and LNs [56,57]. In the tumor microenvironment, PD-L1 and PD-L2 expressed on the surface of tumor cells combine with PD-1 on the surface of T cells, which causes T-cell exhaustion and immune escape of tumor cells [58]. In LNs, PD-L1 and PD-L2, which are highly expressed on the surface of DCs, can also bind to PD-1 on T cells to inhibit their activation. Based on this, Kim et al. [59] integrated the extracellular domain of PD-1 with the C terminus of the H-chain subunit of hFTN, which resulted in the presence of PD-1 on the surface of ferritin nanocages (the new recombinant ferritin named PdNC) for dual targeting of DCs and tumor cells. TEM showed that PD-1-decorated ferritin was still spherical, with a diameter of approximately 20 nm. Compared with those of sPD-1, PdNC exhibited decreased equilibrium dissociation constants (1057 times lower for PD-L1 and 647 times lower for PD-L2), thereby providing increased antagonistic efficiency. At 1 h after intratumoral injection, PdNC effectively reached tumor-draining LNs and gradually accumulated. Evaluation of antitumor immune responses showed that PdNC treatment induced an increase in costimulatory molecules (CD40 and CD86) on DCs and CD44 in CD8^+^ T cells, thereby promoting DC maturation and T-cell activation. In vivo, PdNC significantly increased tumor infiltration of CD8^+^ T cells (by 3.64 times versus the PBS control group), which in turn significantly inhibited the tumor volume (by 75%). Even more surprisingly, approximately 33% of the tumors completely regressed.

Indoleamine 2,3-dioxygenase (IDO) is another immune checkpoint that is frequently implicated in tumor immunosuppression [60]. IDO is a monomeric heme-containing enzyme that is frequently upregulated in cancer cells and host APCs [61]. IDO overexpression leads to the depletion of tryptophan, which is critical for the survival and function of effector T cells; as a result, these cells undergo G1 cycle arrest and apoptosis [62,63]. Furthermore, a metabolite of the enzymatic reaction, kynurenine, can promote the differentiation and activation of regulatory T cells and the recruitment of myeloid-derived suppressor cells to form a therapy-resistant tumor microenvironment. Whereas PDT induces immunogenic cell death, it also promotes IDO expression. Therefore, the combination of an IDO inhibitor and PDT is expected to alleviate PDT limitations and induce stronger antitumor immunity. Yang et al. [64] first encapsulated the photosensitizer ZnF16Pc into hFTN via pH-mediated disassembly methods. Then, composite core/satellite NPs were prepared by covalently coupling ZnF16Pc-loaded hFTN to the surface of NLG919-loaded PEG-PLGA NPs through esterification. DLS showed that the hydrodynamic size was 144.0 ± 1.3 nm. At pH 7.4, in vitro release of NLG919 was sustained, and the cumulative release amount within 24 h was 77.5%, whereas the photosensitizer was hardly released. In vivo, a single treatment inhibited the tumor only initially (ZnF 16 Pc group) or even failed to inhibit the tumor growth (NLG919@PLGA group), whereas the use of composite core/satellite NPs resulted in significant tumor inhibition (84.19% on day 12). Approximately 30% of mice exhibited complete tumor eradication and were still alive after 6 months. A rechallenge experiment demonstrated that composite NPs significantly increased the population of central (CD3^+^CD8^+^CD62L^+^CD44^+^) and effector memory (CD3^+^CD8^+^CD62L^−^CD44^+^) T cells, contributing to strong antitumor immunity.

## 3. Conclusions

In the past decade, hundreds of nanodrug delivery systems have been developed based on passive and active targeting by ferritin, including physical or chemical loading of various therapeutic drugs [8,65] (curcumin [66,67], doxorubicin [68,69], paclitaxel [70,71], and quercetin [72], among others), imaging contrast agents (Fe_3_O_4_ [73,74,75], CuS [76], and Ag [77,78]), and nucleic acids [79]. In this paper, we reviewed the construction and application of ferritin nanodrug delivery systems for tumor immunotherapy in recent years. Through its natural targeting function or by displaying various binding peptides on the surface, ferritin can target tumor cells, tumor-associated fibroblasts, DCs, or TAMs. It can also remodel the tumor immunosuppressive microenvironment and activate inherent and adaptive immunity, thereby achieving long-lasting immune memory and antitumor effects. Based on the cited articles, there are several points that can be optimized during the research process. (1) Clarify the reason for ending the experiment (end point) in antitumor experiments. Animal ethics stipulate that the tumor weight of mice should be less than 10% of their body weight. If the tumor diameter of mice is greater than 1.5 cm (some ethical institutions stipulate greater than 2.0 cm), the experiment should be ended to ensure animal ethics. (2) For ferritin-based tumor immunotherapy, when setting up the control group, an added mixture of antigen and ferritin should be considered and used to compare with the antigen-expressed ferritin via genetic engineering. In this way, we can further confirm what kind of binding mode between antigen and ferritin is more reasonable. (3) For the dosage of positive control, the clinical human dosage and frequency should be taken as the reference. The animal dosage could be calculated through the conversion of body surface area, which is conducive to the transformation in the future.

Notably, a ferritin-based vaccine that promises to protect against multiple coronaviruses by targeting multiple variants of SARS-CoV-2 has entered phase I clinical trials [80]. Joyce et al. [81] linked the ectodomain (residues 12 to 1158) of the SARS-CoV-2 spike protein to the C-terminal region of *Helicobacter pylori* ferritin. After recombinant expression, the vaccine was prepared with liposomes loaded with synthetic monophosphoryl lipid A and saponin QS-21 as an adjuvant. The prepared vaccine elicits high titers of antibodies that neutralize and rapidly protect against SARS-CoV-2 infection after inoculation in non-human primates.

Although ferritin-based formulations have flourished in the past decade and ferritin vaccines have entered clinical trials, some key challenges need to be urgently addressed to move ferritin formulations one step closer to a new generation of nanomedicines. The detailed challenges are listed in the Table 2.

In conclusion, ferritin-based drug delivery holds great potential in tumor immunotherapy, with continuous optimization.

## Figures and Tables

**Table 1:** The key advantages of ferritin-based clinical transformation.

Advantage	Reasons
Good biocompatibility	Ferritin is an iron storage protein that widely found in various living organisms (plants, amphibians, mammals, and others) [23]. The composition of ferritin in mammals is similar and includes an H chain and an L chain [24]. Therefore, ferritin has a high biocompatibility based on its endogenous homology [25]. Using ferritin as a carrier for drug delivery has almost no toxic side effects.
High thermal stability and acid and alkali resistance for easy production	Protein purification often requires complex procedures to separate the target protein from other host cell proteins. Ferritin can withstand a wide range of pH values and temperatures as high as 75 °C for 10 min while still maintaining its icosahedral structure [26]. Therefore, the isolation of ferritin can be carried out using a simple, one-step heat treatment that denatures more than 80% of host cell proteins [27,28]. The excellent physical and chemical properties of ferritin greatly simplify the production process, requiring no special handling during transportation and storage, which distinguishes this protein from many other protein-based drug delivery vehicles and contributes to its clinical translation.
Natural cell-targeting ability	The residues Q14, D15, E17–A19, N21, and R22 in the N-terminal region of the A helix of the H-chain subunit can interact with several residues (R79, F81, Q83, K86, and K87) in the BC loop (short-loop region between the B and C helices) to achieve specific binding of ferritin to the transferrin receptor (TfR) [29].TfR1 is an important transmembrane glycoprotein that regulates cell growth and is expressed on the surface of endothelial cells, erythrocytes, and other cells [10]; however, it is overexpressed in proliferating cells, which require more iron. Thus, TfR1 is widely overexpressed in antigen-presenting cells (APCs) in many malignant tumors, as well as tumor cells. The density of TfR1 on the surface of proliferating cells is approximately 100-fold higher than that in nonproliferating cells. When the density of TfR1 on the cell surface is high, the formed ferritin–TfR1 complex is internalized into intracellular lysosomes for targeted delivery [30]. Thus, H-chain-enriched ferritin is able to target a broad range of tumors, with 10-fold greater potency than that due to the enhanced permeability and retention effect alone, providing a pathway for drug delivery and diagnostic therapy [26]. When the density of TfR1 on the cell surface is low, the formed ferritin–TfR1 complex does not enter intracellular lysosomes to achieve transmembrane transport [31]. In addition, mouse and hFTns are able to interact with mouse T-cell immunoglobulin and mucin domain 2 [26].The L-chain subunit of ferritin has been shown to bind to scavenger receptor class A member 5 (SCARA5), a newly discovered class A scavenger receptor protein. SCARA5 function has not been fully defined, but its expression is closely related to tumor growth and development [32]. Ferritin from horse spleen is a commonly used natural ferritin, consisting of 90% L-chain subunits and 10% H-chain subunits. Studies have found that it can accumulate in large amounts at tumor sites, possibly due to the L chain. This subunit binds to mouse SCARA5 but not to mouse TfR1 [33].
Easily modifiable surface	Each of the 24 subunits of ferritin has amino, carboxyl, sulfhydryl, and other active groups that can be modified by chemical methods, and the amino acid sequence of ferritin can be precisely modified using biological methods. A study showed that amino (3 ± 0.3 lysine residues) and carboxyl (7.1 ± 0.7) groups can be chemically modified on each subunit of the iron-rich ferritin derived from horse spleen [34]. When the iron core is removed, sulfhydryl (derived from 1.0 ± 0.1 cysteine), amino (4.4 ± 0.4 lysine residues), and carboxyl (11.0 ± 0.4) groups can be chemically modified on each subunit of horse-spleen-derived apoferritin [34]. The C-terminal and surface-loop regions of each subunit may provide sites for the insertion of various types of antigenic peptides and small protein antigens.
Small particle size	In normal tissue, the microvascular endothelial space is dense and structurally complete; thus, macromolecules and lipid particles cannot easily penetrate the blood vessel wall. Meanwhile, in solid tumor tissue, the structural integrity of abundant blood vessels is poor, which results in nano-sized openings between microvascular endothelial cells. The lymphatic return in solid tumors is missing. Consequently, drugs can selectively accumulate in tumors because of the enhanced permeability and retention effect. Ferritin is a nanocage with an inner diameter of 8 nm and an outer diameter of 12 nm. The appropriate particle size facilitates its entrance to the target site through the opening of inflammatory microvascular endothelial cells and deep penetration into the tissue.
Hydrophilic channels and the cavity that can be loaded with various drugs	The structure of ferritin, whether it is pure H-chain ferritin, pure L-chain ferritin, or mixed H- and L-chain ferritin, can be disassembled into its various subunits under extremely acidic (pH 2) or alkaline (pH 12) conditions [35]. Then, it reassembles without any external force when the ambient pH returns to the physiological range [35]. This shape-memory capability allows the encapsulation of various types of drugs in the ferritin cavity or the structure serves as a template to mineralize various metal oxides for disease diagnosis and treatment.

**Table 2:** The key obstacles for ferritin-based clinical transformation.

Challenge	Reasons
Standardization of drug loading	Intact hollow, spherical apoferritin is stable in the pH range of 3.40–10.0 [28,82]. As the pH is lowered from 3.40 to 0.80, apoferritin undergoes a gradual breakdown, first forming a hollow sphere with two pores, then a head-mounted structure, and finally a rod-like oligomer. As the pH is increased from 1.96, the disassembled rod-like oligomers first revert to an earphone-like structure and then to a hollow, spherical structure with two-hole defects. When pH continues to increase to neutral or slightly alkaline values, the hollow spherical structure with the two-hole defect still fails to heal [8]. This may affect the homogeneity of ferritin nanocarriers and their in vivo behavior after administration. Therefore, more reliable drug loading methods are needed to obtain defect-free ferritin formulations.
The modification site of ferritin [12]	Modifications at different sites in the ferritin sequence result in different display states of polypeptides or antigens, which may be stretched or gathered and may also affect the natural self-assembly ability and targeting function of ferritin [28,83,84,85]. Therefore, irrespective of whether a chemical modification or gene recombination is used to load antigens or peptides, more investigation is needed on the modification sites.
Animal models	At present, the animal models used to test ferritin-based tumor immune preparations are mainly mice, and the tumor microenvironment is quite different from the clinical reality. Therefore, it is necessary to develop antitumor models in larger animals to simulate actual clinical conditions, especially for the study of antitumor vaccines.
